# Health workforce and governance: the crisis in Nigeria

**DOI:** 10.1186/s12960-017-0205-4

**Published:** 2017-05-12

**Authors:** Davies Adeloye, Rotimi Adedeji David, Adenike Ayobola Olaogun, Asa Auta, Adedapo Adesokan, Muktar Gadanya, Jacob Kehinde Opele, Oluwafemi Owagbemi, Alexander Iseolorunkanmi

**Affiliations:** 1 Demography and Social Statistics, and the e-Health Research Cluster, Covenant University, PMB 1023 Ota, Ogun State Nigeria; 2 Centre for Global Health Research, Usher Institute, University of Edinburgh, Edinburgh, United Kingdom; 3 Department of Surgery, Obafemi Awolowo University Teaching Hospitals Complex, Ile-Ife, Nigeria; 4 Department of Nursing, Obafemi Awolowo University, Ile-Ife, Nigeria; 5 School of Pharmacy and Biomedical Sciences, University of Central Lancashire, Fylde Road, Preston, PR1 2HE United Kingdom; 6 Department of Medicine, Queen Elizabeth University Hospital, NHS Greater Glasgow and Clyde, Glasgow, United Kingdom; 7 Department of Community Medicine, Aminu Kano Teaching Hospital, Bayero University, Kano, Nigeria; 8 National Centre for Technology Management, Obafemi Awolowo University, Ile-Ife, Nigeria; 9 University Health Centre, Covenant University, Ota, Nigeria

**Keywords:** Health workforce, Health crisis, Health governance, Health system, Nigeria

## Abstract

**Background:**

In Nigeria, several challenges have been reported within the health sector, especially in training, funding, employment, and deployment of the health workforce. We aimed to review recent health workforce crises in the Nigerian health sector to identify key underlying causes and provide recommendations toward preventing and/or managing potential future crises in Nigeria.

**Methods:**

We conducted a scoping literature search of PubMed to identify studies on health workforce and health governance in Nigeria. A critical analysis, with extended commentary, on recent health workforce crises (2010–2016) and the health system in Nigeria was conducted.

**Results:**

The Nigerian health system is relatively weak, and there is yet a coordinated response across the country. A number of health workforce crises have been reported in recent times due to several months’ salaries owed, poor welfare, lack of appropriate health facilities and emerging factions among health workers. Poor administration and response across different levels of government have played contributory roles to further internal crises among health workers, with different factions engaged in protracted supremacy challenge. These crises have consequently prevented optimal healthcare delivery to the Nigerian population.

**Conclusions:**

An encompassing stakeholders’ forum in the Nigerian health sector remain essential. The national health system needs a solid administrative policy foundation that allows coordination of priorities and partnerships in the health workforce and among various stakeholders. It is hoped that this paper may prompt relevant reforms in health workforce and governance in Nigeria toward better health service delivery in the country.

## Background

Across several low- and middle-income countries (LMICs), national health systems have been functioning sub-optimally due to recurring challenges within and external to the health sector, with this particularly affecting the delivery of accessible and affordable healthcare services [1]. The health system, in most cases, is synonymous to publicly owned health facilities, with several important private and non-state actors being downplayed [2]. The functional capacities of the health system in these settings have gradually weakened, having failed to recognize and maximize efforts of all organizations, institutions, structures and resources primarily devoted to improving health.

The health workforce—all persons involved in activities primarily devoted to enhancing health—is an essential block of any functioning health system in any country, in the absence of which clinical and public health services cannot be delivered to the population [3]. Health governance is the administrative umbrella of the health system primarily concerned with policymaker- or government-led steering and rule-making functions targeted at achieving national health policy objectives for effective delivery of health services and attainment of universal health coverage [4]. Experts have actually shown direct links between efficient health system governance and promising health workers outputs, which ultimately have positive effects on overall health outcomes [2]. However, poor administration and continued underinvestment in health, even with the spread of HIV/AIDS, re-emerging diseases and persistent violent conflicts, have contributed greatly to the fragile health systems in many African states [5, 6]. While there have been calls for concerted efforts—social, economic, environmental and multisectoral—toward health system strengthening in sub-Saharan Africa (sSA) [6], human resources’ crises in the health sector have continued to be a major challenge [7]. The governments of many African countries have reported challenges in training, funding, employment, capacity building and efficient deployment of the health workforce [7]. Consequently, the continent has continued to experience a rapidly progressive health workforce migration to high-income settings in search of better opportunities [8].

Crises within the health workforce have been reported as perhaps the biggest constraint towards global health system development and sustenance, particularly in Africa [8]. According to the 2006 World Health Report, 57 countries were in severe health workforce crises, with 37 of these in sSA—a region with only 3% of global health workforce, despite contributing about a quarter to the global disease burden [3]. Nigeria, the most populous African country, possibly contributes even more to these crises in the region. Increasing annual rates of population growth, as observed in Nigeria, has been adjudged a major factor in countries with severe health workforce crises [9]. Nigeria obviously requires significant increase in the number of additional health workers to achieve desired population coverage. However, beyond the shortfall in health workforce, the Nigerian health sector has particularly experienced a number of other lingering crises in recent times. There are growing concerns locally and internationally over these issues, with this linked to the overall poor states of health governance in the country [10]. This paper therefore provides insights into a rapidly changing developing economy with a weak national health system governance and shortage of human resources for health. We examined some major health workforce crises in the Nigerian health sector in recent years (2010–2016), to understand key underlying causes and provide feasible recommendations toward preventing and/or managing potential future occurrences in the country.

## Methods

We conducted a scoping literature search of PubMed on 10 August 2016 to identify recent studies (2000–date) on health workforce crisis and health governance in Nigeria. Using Boolean operators ‘OR’ and ‘AND’, relevant Medical Subject Headings (MeSH) terms were combined (Table 1). Google Scholar and reference lists of selected studies were further searched to identify more studies. Due to several media hits on Google Scholar, we also searched for newspapers articles and contacted some authors of related publications for further clarifications.

**Table 1:** Search terms on PubMed

Number	Searches	Hits
1	doctors strike	507
2	health crisis	17 367
3	health workers	130 368
4	health workforce	56 412
5	(((doctors strike) OR health crisis) OR health workers) OR health workforce (i.e. *1 OR 2 OR 3 OR 4*)	155 211
6	health governance	6 756
7	health administration	893 315
8	health leadership	29 657
9	health system	382 465
10	(((health governance) OR health administration) OR health leadership) OR health system (i.e. *6 OR 7 OR 8 OR 9*)	1 174 194
11	Nigeria	39 810
12	((((((doctors strike) OR health crisis) OR health workers) OR health workforce)) AND ((((health governance) OR health administration) OR health leadership) OR health system)) AND Nigeria (i.e. 5 AND 10 AND 11)	515

To minimize biases in the selection of studies and ensure a balanced presentation of the findings and discussion, the authors were invited from various health sectors and relevant backgrounds in Nigeria. These include epidemiologist (DA), surgeons (RAD, OO), nurse (AAO), pharmacist (AA), physician (AdA), public health physician (MG), librarian and health informatician (JKO), general practitioner (AI), academics (DA, AA, MG) and health sector leader (OO). Data were double extracted by DA and RAD and checked by all other authors for consistency. From each study (or media hit), we broadly checked for reported industrial actions (or crises) within the Nigerian health sector from 2010 upwards, the health workforce involved, the specific period covered and reasons for such crises. We classified identified events into national and regional industrial actions, respectively (Table 2). From the reasons reported, we extracted themes and presented an over-arching discussion on the recent health workforce crises (2010–2016) and the health system in Nigeria. Using the World Health Organization (WHO) standards and applicable contextual factors, we provided relevant recommendations for changes in health system governance in Nigeria. Ethical approval was not required for this study, as this was a review of publicly available literature, information and data.

**Table 2:** Selected health workforce industrial actions in Nigeria, 2010–2016

Type of industrial action	Health workforce	Period	Reason	Themes identified
National	Joint Health Sector Unions (JOHESU)^a^	July 2014	That members be made consultants like medical doctors; demand to establish directorates for nursing, pharmacy, physiotherapy, and other allied health sectors; request for an amendment bill to correct marginalization of all health workers by doctors composition and appointment of the management boards of health institutions; extension of retirement age from 60 to 65 years; implementation of the National Health Insurance Scheme towards increased remuneration and overall funding of health system	Administration, leadership, governance, policy, finance, remuneration, supremacy challenge
	Nigerian Medical Association (NMA)	July–August 2014	Press for relativity^b^ and skipping^c^ in doctors salaries; to reverse the consultant status and directorates of allied health professionals; call for improved funding of health system	Administration, funding, remuneration, supremacy challenge
	National Association of Resident Doctors (NARD)	2011, 2013 and 2016	Call for teaching allowance and skipping; upgrade of doctors to Integrated Payroll and Personnel Information System (IPPIS) platform; full implementation of adjusted Consolidated Medical Salary Structure (CONMESS) across board; request for residency training guidelines, appraisal and upgrading; request for Federal Government to address high-handedness of chief medical directors of some health institutions; implementation of the National Health Act	Administration, governance, policy, funding, remuneration
Local	NMA Lagos chapter	2013	Request for improved conditions of service, better welfare and improved facilities	Welfare, funding
	Association of Resident Doctors (ARD)—selected local hospital chapters	2010–2016	Mainly protests over actions of chief medical directors (CMDs) including irregular and non-payment of salaries for several months, poor welfare (demand for renovation of call rooms and improved call meals), non-payment of teaching allowances and update courses and shortage of doctors in the hospitals, as interns and residents completing training were not promptly replaced	Administration, leadership, health workforce distribution, welfare

^a^JOHESU consists of five registered health professionals unions: Medical and Health Workers’ Union of Nigeria (MHWUN), National Association of Nigeria Nurses and Midwives (NANNM), Senior Staff Association of Universities, Teaching Hospitals, Research Institutes and Associated Institutions (SSAUTHRIAI), Nigeria Union of Allied Health Professionals (NUAHP) and Non- Academic Staff Union of Educational and Associated Institutions (NASU)

^b^Relativity implies maintaining a differential remuneration between doctors and other health workers [16, 20, 22, 24]

^c^Skipping implies the skipping of grade level 12 which was first effected for non-doctor health workers [16, 20, 22, 24]

## Results

### Search results

Our PubMed search returned 515 studies (Table 1). We selected 12 studies [11–22] that broadly described health workforce crises in Nigeria. Four other sources of information were identified from further searches, with three of these being media hits [23–25], and the fourth is a needs assessment of the Nigerian health sector [26].

### Case presentation: The Nigerian health workforce crisis

#### Distribution of health workforce

Persistently low and inequitable distribution of health workers remain a major challenge in the Nigerian health sector [11]. Bangdiwala et al. stated that there is a global crisis in health workforce as shown by acute shortages and uneven distribution of health workers in several settings [12]. In Nigeria, experts have reported that historically, ‘*brain drain*’, in the form of migration of health workers to high-income settings, has been a major setback in the country [13]. Current statistics show that 1 in 4 doctors, and 1 in 20 nurses, trained in Africa are currently working in developed countries, with this accounting for a shortfall of over 1.5 million health workers in the region [4]. In Nigeria, the health workforce density is estimated at 1.95 per 1000 population [10]. Abimbola and colleagues reported that this shortfall in Nigeria has been further complicated by gross inequity in health workforce distribution, as there is no national policy guiding the postings and transfers of health workers—redeployment is often based on discretion of administrative officers with multiple influences and several competing interests [11].

#### Remuneration and welfare

Poor remuneration and welfare of the health workforce have also been reported [14]. Health workers have embarked on industrial action due to several months’ salaries owed and poor working conditions (Table 2). In a recent survey of senior management staffs of health institutions in Nigeria, massive discrepancies in remuneration of health workers on the same grade levels across the federal, state and local governments were observed [26]. In some health centres, very difficult working conditions, mostly due to the lack of power supply and poor call rooms and meals, have been described [14]. Moreover, several health facilities are grossly under-supplied. There are no provisions for regular clinical training across several health centres, despite credit units needed from this for continuing professional development, which is mandatory for renewal of practicing license [14].

#### Supremacy challenge

The health workforce crises in the country have taken quite unique, yet worrying, dimension in recent times (Table 2). Distrust, dissensions and recurring conflicts among different professional groups in the health sector are now emerging [23]. Other health workers have alleged that the Nigerian health system is designed to favour doctors mainly [15]. The alleged dominance of doctors over the years has encouraged other health workers to form the new group called the Joint Health Sector Unions (JOHESU) [15]. Disputes over salaries, allowances, consultancy status and who heads the health sector have continued to emerge among different factions. For example, the Nigerian Medical Association (NMA) and JOHESU strikes in 2014 were based on doctors versus nurses, doctors versus pharmacists, doctors versus laboratory workers and doctors versus other allied health professionals protracted supremacy challenge [23].

#### Ph.D. versus Fellowship

Recently, crises based on qualifications have also been reported among medical and non-medical academics—issues bordering on whether a Ph.D. or a Fellowship of the postgraduate medical colleges is required for an academic appointment [16]. Onwudiegwu emphasized that medical expertise does not necessarily imply effective teaching skills; as such, all medical school teachers without formal teaching and pedagogical training need to enrol for a Ph.D. to continue to hold academic positions in teaching hospitals [16]. Many have challenged and raised serious concerns about this within the health sector, particularly because there are no Masters/Ph.D. programmes for most of the clinical specialties in Nigeria, suggesting this may possibly be another dimension to the existing supremacy issue. This therefore remains an ongoing challenge in the delivery of expected teaching and core health services among concerned professionals.

### Case presentation: The Nigerian health system leadership

The central themes emerging from Table 2 are ‘*administration*’, ‘*leadership*’ and ‘*governance*’, as most of these industrial actions resulted from poor administration in the hospitals and failure of the government to decisively respond. The government often fail to address core issues until the health workers resort to protests. This is in keeping with a recent study by Oleribe et al. who clearly cited poor healthcare leadership and management as the most common and most important factor attributable to industrial actions by Nigerian health workers [14].

Even amidst government interventions, promises and responses are often focussed on the immediate—to make striking workers resume duties—only to renege on such promises after resumption. The massive sack of striking health workers and policy of ‘no work no pay’ adopted by the federal and many state governments were also attempts to arm-twist the workers into resumption without really addressing the underlying issues. For instance, Odigwe noted that the government of Lagos State in Nigeria in 2012 could only respond to an indefinite strike over non-payment of salaries by sacking nearly 800 doctors on the grounds that they were insensitive to patients’ needs [17].

In some instances, these industrial actions were not always due to the government’s negligence. As observed in Table 2, some chief medical directors (CMDs) chose not to pay health workers’ salaries and maintain health facilities even when funds may have been disbursed for this purpose. Besides, the introduction of the various electronic payment platforms, such as the Integrated Payroll and Personnel Information System (IPPIS), may have further affected early payment of salaries due to technical and operational challenges of the electronic systems [24]. It is also possible that the government is yet to critically appraise the issues raised by the health workforce who are sometimes relatively better informed on the major challenges within the health sector. Rather, the government has often leveraged on the public notion that health workers should not go on strike, thereby employing the media to fight back the protesting workers. In fact, a Marxist interpretation has long been given to the actions of doctors in Nigeria, suggesting they are a powerful and privileged group, who leverage on this to pursue their personal interests [18]. One classic example is that doctors tend to benefit most from repeated strike actions, as patients are left with no option than to patronize private hospitals, which are mostly owned by the striking doctors [25]. While this may relatively be a fact, it is also important to emphasize that the failure of the government to effectively manage prevalent health crisis actually led to the establishment of more private hospitals in the country. Private clinics have been complementary to existing government health facilities, as such clinics are often close to the people and are considerably affordable. An engaging and encompassing health system governance framework involving both public and private sectors is needed to achieve population health objectives [6].

## Discussion

According to the WHO, a strong health system has a robust finance structure, well-remunerated and trained workforce, sufficient and highly maintained facilities, logistics for medicines, vaccines and technologies and a reliable and regularly updated health information system [27]. With appropriate governance, all these ensure accessible and timely health service delivery to the population [27]. As demonstrated by the repeated workers’ protests, relatively poor state of health facilities, sub-optimal management of common diseases and high rates of medical tourism, among many others, the Nigerian health system is seemingly not fulfilling her obligations. Indeed, the recent democratic changes and pressure from social media campaigners have ensured sustained pressure on the Nigerian government to improve health service delivery. Provisions of free or improved primary healthcare services have been major contents of various electoral manifestoes in the country since the return to civilian rule in 1999. Looking back, however, several population health challenges remain. There are only few well-equipped and appropriately staffed government-run primary, secondary and tertiary health facilities in Nigeria to meet population-wide health needs [26]. The private sector has actually been a major stakeholder in health service delivery in the country. For example, most kidney transplants in Nigeria have been conducted in private facilities [28]. However, there are concerns that the activities of several private facilities are not regulated and fall short of the standard management guidelines for many diseases [10].

Current health statistics in Nigeria point to the deteriorating capacity of the health system, with an estimated infant mortality rate at 69/1000 live births; under-five mortality rate at 109/1000 live births; maternal mortality ratio at 814/100 000 live births; average life expectancy for men and women at 53 and 56 years, respectively; and relative probability of dying between 15 and 60 years estimated at 341 per 1000 population [10]. In the last 10 years, there have been calls to address these prevailing issues, especially on the provision of better facilities for disease diagnosis and treatment, improved health workforce welfare and remuneration and a national health insurance scheme. One notable response is the National Health Act, which was signed into law by the former President Goodluck Jonathan on October 31, 2014, albeit having generated diverse disagreements and interests among various health professionals and stakeholders in the preceding 5 years [19]. This is not unexpected as Nigeria actually operates a pluralistic healthcare system with the orthodox and traditional health practitioners operating without any collaboration, and the latter particularly having no regulation [26]. Moreover, both private and public sectors provide the orthodox health services, with the private contributing over 65% of services despite owning only 38% of health facilities [26]. Many of the stakeholders in the health sector have also benefitted from the continued underinvestment in health, due to massive funds and aids received from international agencies and donors [19]. All these have contributed to various interests among different stakeholders in the health sector, leading to the protracted delays with the formal signing of the Act. The Act is made of seven parts which establishes a legal framework for the regulation, development and management of the Nigerian health system and also set standards for rendering health services in the country [29]. Each part contains essential sections towards positively impacting universal health coverage, healthcare cost, healthcare access, practice by healthcare providers, quality and standards, patient care and health outcomes. When fully implemented, it is equally expected to address funding and insurance facilities, including free medical care for under-fives, pregnant women, and elderly people with disabilities [29]. However, it is still not surprising that more than 2 years after being signed into law, the Act is yet to be implemented. In fact, a recent report revealed that despite being aware of the Act, the overall knowledge of Nigerian health professionals on the Act is generally poor [19]. Due, in part, to non-implementation of the Act, the National Health Insurance Scheme (NHIS) could only achieve 4% coverage since it was launched 12 years ago [20], although difficulties in convincing individual state governments to launch the scheme and the lack of adequate health workforce to potentially meet increased demand may have also contributed to non-implementation at the state and local government levels, respectively [20]. As such, private out-of-pocket-expenditure on health still accounts for over 70% of the estimated per capita expenditure on health in the country, with this affecting equitable access to quality and affordable healthcare delivery [26].

Meanwhile, there are occasions when the Nigerian government demonstrated her capacity to adequately and rapidly respond to population health needs despite the obvious challenges in the health sector. For example, during the recent Ebola virus disease outbreak in West Africa, the coordinated response in Nigeria was due mainly to the administrative and financial strengths of the then governments of Lagos and Rivers states—the two states affected in the country—with necessary overall support of the Federal Government. The relative success with the Polio Eradication Programme in the country is another basic example. Experts have stated that in both instances—Ebola and polio, the impressive use and coordination of emergency operation centres (EOCs) must be credited for the successes recorded [30]. This received strong political commitment, population willingness, strategic operational support and great deal of accountability [30]. Invariably, this completely contradicts the idea of the government’s lack of capacity to respond to population health needs and implement and sustain interventions. It perhaps implies some selective response to specific health issues by the government, possibly to those issues having high potential to attract international media and accolades. The Nigerian health workforce may have been frustrated overtime, knowing fully well that there are enough resources to respond to basic health needs in the country, with this resulting in the numerous protests and demonstrations against the government in recent times. Several health systems have failed mostly due to their lack of resilience and capacity to respond to pressing health workforce and larger population health needs [31].

### Way forward

Based on our findings, it is evident that health workforce crises in Nigeria have continued to deteriorate due to some specific factors, mostly related to poor health leadership. As a way forward, some basic questions need to be addressed—Who benefits from industrial actions? How can conflicts among health professionals be addressed? How can negotiations be initiated? What solutions are needed and who should implement these? Who are the prominent actors? How can health leadership be built? To address these questions, an open stakeholders’ conference is important (and possibly on a regular basis) where all can agree on the essential challenges of the health sector, develop practical solutions and jointly work towards strengthening the health system. The public needs to clearly understand the grievances of the health workers, especially towards the government. No doubt, the health workers directly benefit from government’s interventions, but the public benefits even more when health service delivery is optimal.

To build sustainable leadership, it is important the national health system has a solid administrative policy foundation that allows alignment, coordination and coherence of priorities and partnerships in the health workforce and among various stakeholders [6]. The mutual distrust, tension and supremacy challenge among the health workforce need to stop as a matter of priority. The focus of health service delivery should be on teamwork, rather than factional or individual strengths. Many have argued that this is perhaps the single most important factor in Nigeria, and until agreeable solutions are found across various professional groups, the Nigerian health system may continue to suffer from repeated and avoidable disruption of health service delivery [21].

To address the challenges in medical training and academic appointments, it has been suggested that current residency programmes (postgraduate medical training) in Nigeria be structured and upgraded to allow for both academic and clinical training as observed in some other countries, such that residents are awarded Masters or Ph.D.s, along with their respective Fellowships, on completing their training [16]. Moreover, there is a need to design a contextually adaptable framework for inter-professional education and collaborative practice in the health sector as recommended by the WHO, to further facilitate successful cooperation, communication and teamwork in health service delivery and ensure a health workforce that is better prepared to respond to local health needs [32]. The goal of the health system is to ensure delivery of affordable, accessible, equitable and safe health services to the population, and in achieving this, every health worker has an important role to play.

Lack of funds, inadequate welfare and poor state of health facilities have been major factors affecting workers’ motivation. While these need to be addressed urgently, there is also the need to strategically adopt broader range of motivational factors [22]. Some authors have suggested that one basic way to address conflicts among health professionals and possibly stem industrial disputes is to introduce an evenly spread non-financial benefits to the health workforce [21]. This may be in form of career development, special skills acquisition and other varieties of human capacity development programmes. The existing NHIS, which still covers only the formal sector, albeit marginally, needs to be restructured and strengthened to facilitate universal free health coverage for all Nigerians, learning from the National Health Service (NHS) scheme in the United Kingdom of Great Britain and Northern Ireland and other functional insurance schemes in developed countries. In the NHS for instance, health care is provided by the government and funded by the taxpayer [33]. Hence, with the exception of some specific charges, consultations and treatments remain free at the point of use for all UK residents [33].

Meanwhile, a dedicated and sustainable tax scheme is also needed to generate funds for the Nigerian NHIS. As there are also issues with mismanagement of funds in the country, credible health economists or financing agencies (local and international) can be engaged in a public-private partnership, and corporate bodies may also be incorporated into the scheme to increase efficiency, productivity and accountability. To maximize use of funds, health research can be directed towards low-cost technology rather than expensive high-tech medical equipment, which requires extensive human, technical and financial resources to maintain. With responsible and committed leadership and relative improvement in population health indicators, human, technical and financial assistance can be requested from external multi-national organizations to sustain the potential positive changes. It is important to note that this paper is not in any way exhaustive of the problems in the Nigerian health sector, as there are still several issues, such as those related to government planning for health workers, mix of cadres in the health sector, capacity of specialist disciplines and re-defining primary versus specialist care, that are subject to further deliberations, research and policy reforms.

## Conclusions

Good governance is needed to achieve a sound national health system, especially with regard to human resources for health. The Nigerian health system is lacking full capacity in leadership and governance, with this reflecting in the health workforce crises and poor health service delivery in recent years. Although the Nigerian government can be responsive to population health needs, without a driving, visionary, systemic and structural change in health governance, the prevailing crises in the health workforce and service delivery may continue. According to Dr Jong-Wook Lee, former Director General of the WHO, there is an urgent need to work together towards ensuring access to a motivated, skilled and supported health worker by every person everywhere [3]. We hope this paper can prompt appropriate debate, deliberations and changes in health workforce and governance in Nigeria toward better health service delivery in the country.

## Abbreviations

ARD: Association of Resident Doctors
CMD: Chief medical director
EOCs: Emergency operation centres
JOHESU: Joint Health Sector Unions
NHIS: National Health Insurance Scheme
NHS: National Health Service
NMA: Nigerian Medical Association
sSA: Sub-Saharan Africa
WHO: World Health Organization

## References

[1] Singh P, Sachs JD (2013). 1 million community health workers in sub-Saharan Africa by 2015. Lancet.

[2] World Health Organization (2007). Everybody’s business: strengthening health systems to improve health outcomes: WHO’s framework for action.

[3] World Health Organization (2006). The World Health Report 2006—working together for health.

[4] World Health Organization (2014). Health systems. World Health Statistics 2014.

[5] Chen L, Evans T, Anand S, Boufford JI, Brown H, Chowdhury M, Cueto M, Dare L, Dussault G, Elzinga G (2004). Human resources for health: overcoming the crisis. Lancet.

[6] Senkubuge F, Modisenyane M, Bishaw T (2014). Strengthening health systems by health sector reforms. Glob Health Action.

[7] Dovlo D (2005). Wastage in the health workforce: some perspectives from African countries. Hum Resour Health.

[8] Dovlo D (2007). Migration of nurses from sub-Saharan Africa: a review of issues and challenges. Health Serv Res.

[9] Pacqué-Margolis S, Muntifering C, Ng C, Noronha S, IntraHealth International (2011). Population growth and the global health workforce crisis.

[10] World Health Organization (2016). Nigeria. Global Health Workforce Alliance.

[11] Abimbola S, Olanipekun T, Schaaf M, Negin J, Jan S, Martiniuk AL: Where there is no policy: governing the posting and transfer of primary health care workers in Nigeria. Int J Health Plann Manage. Int J Health Plann Manage 2016, Epub ahead of print. 10.1002/hpm.2356 PMC5716250 27144643

[12] Bangdiwala SI, Fonn S, Okoye O, Tollman S (2010). Workforce resources for health in developing countries. Public Health Rev.

[13] Ike SO (2007). The health workforce crisis: the brain drain scourge. Niger J Med.

[14] Oleribe OO, Ezieme IP, Oladipo O, Akinola EP, Udofia D, Taylor-Robinson SD (2016). Industrial action by healthcare workers in Nigeria in 2013-2015: an inquiry into causes, consequences and control-a cross-sectional descriptive study. Hum Resour Health.

[15] Alubo O, Hunduh V: Medical dominance and resistance in Nigeria’s health care system. Int J Health Serv 2016, Epub ahead of print. 10.1177/0020731416675981 27793984

[16] Onwudiegwu U (2010). Medical teachers and teaching qualification: abstracts from Nigerian Medical Association Annual Scientific Meeting, April 21–25, 2010. Nigerian Med J.

[17] Odigwe C (2012). Lagos state doctors are sacked for striking over pay. BMJ.

[18] Alubo SO (1986). The political economy of doctors’ strikes in Nigeria: a Marxist interpretation. Soc Sci Med.

[19] Enabulele O, Enabulele JE (2016). Nigeria’s National Health Act: an assessment of health professionals’ knowledge and perception. Niger Med J.

[20] Okebukola PO, Brieger WR (2016). Providing universal health insurance coverage in Nigeria. Int Q Community Health Educ.

[21] Akinyemi O, Atilola O (2013). Nigerian resident doctors on strike: insights from and policy implications of job satisfaction among resident doctors in a Nigerian teaching hospital. Int J Health Plann Manage.

[22] Bhatnagar A, Gupta S, Alonge O, George AS: Primary health care workers’ views of motivating factors at individual, community and organizational levels: a qualitative study from Nasarawa and Ondo states, Nigeria. Int J Health Plann Manage 2016, Epub ahead of print. 10.1002/hpm.2342 27062268

[23] JOHESU press release on the NMA strike and the imminent crisis in the health sector [cited 2016 September 09]; Available from: http://www.medicalworldnigeria.com/2014/07/johesu-press-release-on-the-nma-strike-and-the-imminent-crisis-in-the-health-sector#.V86VjpgrLIU.

[24] Nobody can sack you—JOHESU orders health workers to begin 7-day strike [cited 2016 September 09]; Available from: http://dailypost.ng/2016/06/22/nobody-can-sack-you-johesu-orders-health-workers-to-begin-7-day-strike/.

[25] Strike: JOHESU accuses doctors of short practices [cited 2016 September 09]; Available from: http://www.news247.com.ng/news/strike-johesu-acusses-doctors-of-short-practices.

[26] Omoluabi E (2014). Needs assessment of the Nigerian health sector.

[27] World Health Organization. World Health Report 2010—health systems financing: the path to universal coverage. Geneva: World Health Organization; 2010. 10.2471/BLT.10.078741 PMC2878164 20539847

[28] How private hospitals are championing kidney transplants—urologist [cited 2016 September 09]; Available from: http://www.dailytrust.com.ng/news/health/how-private-hospitals-are-championing-kidney-transplants-urologist/107334.html.

[29] The Senate (2014). National Health Bill 2014.

[30] Shuaib FM, Musa PF, Muhammad A, Musa E, Nyanti S, Mkanda P, Mahoney F, Corkum M, Durojaiye M, Nganda GW (2017). Containment of Ebola and polio in low-resource settings using principles and practices of emergency operations centers in public health. Public Health Manag Pract.

[31] Campbell J, Cometto G, Rasanathan K, Kelley E, Syed S, Zurn P, de Bernis L, Matthews Z, Benton D, Frank O (2015). Improving the resilience and workforce of health systems for women’s, children’s, and adolescents’ health. BMJ.

[32] World Health Organization (2010). Framework for action on interprofessional education and collaborative practice.

[33] Berwick DM (1998). The NHS: feeling well and thriving at 75. BMJ.

